# Development of a family physician impact assessment tool in the district health system of the Western Cape Province, South Africa

**DOI:** 10.1186/s12875-014-0204-7

**Published:** 2014-12-12

**Authors:** Kevin S Pasio, Robert Mash, Tracey Naledi

**Affiliations:** Division of Family Medicine and Primary Care, Stellenbosch University, Box 19063, Tygerberg, 7505 South Africa; Health Programmes, Department of Health, Western Cape Government, Western Cape, South Africa

**Keywords:** Family practice, Family physicians, Physician’s role, Validation studies, South Africa

## Abstract

**Background:**

Policy makers in Africa are ambivalent about the need for family physicians to strengthen district health services. Evidence on the impact of family physicians is therefore needed. The aim was to develop a tool to evaluate the impact of family physicians on district health services according to the six expected roles that have been defined nationally.

**Methods:**

Mixed methods were used to develop, validate, pilot and test the reliability of the tool in the Western Cape Province, South Africa. An expert panel validated the content and construction of the tool. The tool was piloted by 94 respondents who evaluated eight family physicians. Cronbach alpha scores were calculated to test the reliability of the tool. The impact of these family physicians in the pilot study was also analysed.

**Results:**

A draft tool was successfully developed, validated, and proved reliable (Cronbach alpha >0.8). The overall scores (scale of 1–4) were: Care provider = 3.5, Consultant = 3.4, Leader and champion of clinical governance = 3.4, Capacity builder = 3.3, Clinical trainer and supervisor = 3.2 and Champion of community-orientated primary care (COPC) = 3.1. The impact on COPC was significantly less than the impact of other roles (p < 0.05).

**Conclusion:**

The Family Physician Impact Evaluation Tool can be used to measure the impact of family physicians in South Africa. The pilot study shows that the family physicians are having most impact in terms of clinical care and clinical governance, and a lesser impact in terms of clinical training, capacity-building and especially COPC.

**Electronic supplementary material:**

The online version of this article (doi:10.1186/s12875-014-0204-7) contains supplementary material, which is available to authorized users.

## Background

Africa has the world’s highest burden of disease, lowest life expectancy and most scarce human resources for health [1]. In this context, effective primary health care and district health services are seen as one of the essential ingredients for health systems to make a difference [2]. Family physicians are medical doctors that have received postgraduate training to become expert generalists and have been identified as one of the essential members of the healthcare team that are needed to deliver effective primary health care [2,3].

Despite this, Africa is also the continent that has least embraced the training of family physicians. Policy-makers and leaders of the health system in many African countries are unsure about the contribution that family physicians can make and whether they are cost-effective and feasible in our context [4]. Many countries are starting to explore or initiate training programmes in family medicine, but stronger evidence of the contribution of family physicians to health systems in Africa would strengthen progress in this regard [5]. There is therefore a need to generate evidence in countries such as South Africa, which have committed themselves to the training and deployment of family physicians, on what early impact family physicians are having in the health system.

Research in the USA has demonstrated that the supply of primary care physicians is positively associated with better population health and can help tackle the negative health effects of social inequalities [6,7]. Other high income countries such as the UK and Australia are committed to general practitioners within primary care and to the value of medical generalism [8,9]. In middle income countries, such as Brazil, family doctors have been seen as essential members of healthcare teams caring for defined populations [10,11]. Even in low income countries, such as Nepal, family physicians have been shown to make a difference to access to essential services at district hospitals [12].

One of the key issues in Africa has been the development of a more contextualised model of the role and contribution of family physicians [13]. It is clear that the generalists in Africa must work alongside traditional healthcare systems, with limited resources and with many geographical or logistic barriers to referring patients to other levels of care [14]. Most countries have insufficient resources to place family physicians in primary care as the first contact health worker and rely on nurse practitioners or clinical officers. Many countries also make use of community health workers to bring primary health care into homes and communities. In this context family physicians are important in terms of mentoring and capacity-building for the rest of the healthcare team and seeing more complicated patients. In addition family physicians are usually expected to work at the local district hospital and therefore also require a more extended skills set that includes competencies in obstetrics and anaesthetics for example [15].

The South African National Development Plan (NDP) 2030 imagines a future in which South Africa has “a health system that works for everyone and produces positive health outcomes” [16]. It acknowledges the challenges facing South Africa’s health system arising from the legacy of apartheid and the quadruple burden of disease: HIV/AIDS and tuberculosis (TB); maternal and child morbidity and mortality; non-communicable diseases; violence, injuries and trauma [17,18]. It also commits itself to the ongoing re-engineering of primary health care (PHC) and the introduction of National Health Insurance [19], to ensure that “everyone has access to appropriate, efficient and quality health services” [16]. The district health system (DHS) has been identified as the vehicle for the implementation of PHC and family physicians have been designated to “take primary responsibility for developing a district specific strategy and an implementation plan for clinical governance…provide technical support and capacity development for implementing clinical governance tools, systems and processes to ensure quality clinical services…[and] also take overall responsibility for monitoring and evaluating clinical service quality for an entire district” [16].

**Figure 1:**
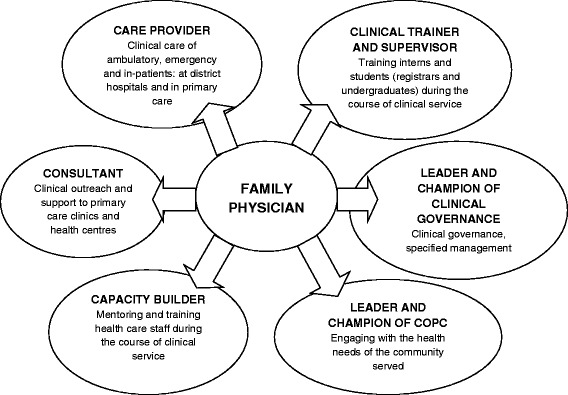
**Roles and competencies expected of a family physician.**

The formal training of family medicine registrars in South Africa only began in 2008, following the promulgation of the new speciality in August 2007 [20]. The first qualified family physicians only graduated in 2011, although there was a body of family physicians previously trained under a variety of older more part-time programmes. The new graduates are specifically trained to work as expert generalists in the district, including the district hospital [21]. Six key roles for the family physician have been agreed to nationally as shown in Figure 1 [22].

The transformation of the health services in the Western Cape Province has included a specific commitment to deploying family physicians within the district health services [22,23]. Since 2011, when the first intake graduated, there has been a steady increase in the employment of family physicians in the Western Cape from 21 in 2011 to 45 in 2014; and an estimated increase to 60–80 over the next five years [22]. Despite this commitment in the Western Cape many other provinces in South Africa remain uncertain or even sceptical as to the roles and impact of family physicians.

Although family physicians are seemingly making a positive contribution to health care in the Western Cape, there is no substantial research evidence to support this [23]. As mentioned above, there are also many in South Africa and the region who are ambivalent about family physicians and their role [24]. The aim of this study therefore was to develop a family physician impact evaluation tool to evaluate the perceived impact of family physicians on health system performance and clinical processes in district hospitals and primary care facilities in the Western Cape.

## Methods

### Study design

Mixed methods were used in the development, validation, piloting and testing of the tool. Validation of the tool involved qualitative feedback on the content and construct from an expert panel. Piloting and testing of the tool involved both qualitative feedback as well as quantitative analysis of the data obtained from the tool and between raters.

### Setting

The tool was developed in the Cape Winelands and Overberg districts of the Western Cape, which is a rural area more than 100 km from Cape Town. The population are mainly poor, uninsured, working on farms and dependent on the public health sector. The districts contain a number of small rural towns, each of which have a district hospital and associated primary care clinics. The area includes one larger town, Worcester, which contains a regional referral hospital and a community health centre. Family physicians have been employed at all of the district hospitals and the community health centre. At the district hospitals the family physicians also support the local primary care clinics. The regional hospital contains a family medicine department with three family physicians, two of whom are also heavily involved in the Rural Clinical School, which is a satellite campus of Stellenbosch University.

### Developing the tool

A draft tool was developed by reviewing the roles of the family physician outlined in the national core standards for training [21,25] and in the Western Cape’s District Health Services policy documents [23]. It was decided that the tool would focus primarily on the key roles of the Family Physician, namely: care provider, consultant, leader and champion of clinical governance, clinical trainer and supervisor, capacity builder, and leader and champion of COPC. A set of statements were developed for each role, based on the researcher’s understanding of what each role entailed. The one researcher was a registrar in family medicine and the other was a family physician and professor of family medicine and primary care at Stellenbosch University. The statements attempted to measure the perceived impact of these roles within the district health services from the perspective of those working alongside the family physician. In this draft tool, respondents could indicate their agreement with these statements by ticking one of four boxes, either: strongly disagree, disagree, agree or strongly agree.

The tool was designed with the following respondents in mind: managers (district, facility and nursing managers), doctors (family physicians, medical officers, community service doctors and interns), nursing staff and other health professionals. The family physicians being evaluated would also be involved in the process by conducting the same evaluation on themselves.

### Validating the tool

Once the draft tool was completed, a selected group of experts was invited to validate the content and construct of the tool. Experts were selected on the basis of being the Family Medicine postgraduate programme managers at the University of Cape Town and Stellenbosch, the six district managers in the Western Cape, the five Family Medicine training complex coordinators in the Western Cape, three other researchers who were also conducting research into the impact of Family Physicians in the Western Cape, and the director of the Health Impact Assessment Directorate for the Western Cape. An email was then sent to this group of experts explaining the research and the tool, and what the validation process would entail. The draft tool was attached to the email, with space below the statements for comments and suggestions. The experts were asked to comment on the content of the tool and on whether anything needed to be excluded or added. They were also asked to comment on the construction of the tool and statements and whether it adequately captured the roles and potential impact. Finally they were asked to make any comment on the layout and appearance of the tool. Feedback from the experts was only obtained once and the tool was then revised based on this feedback.

### Piloting the tool

Following the validation of the tool, the tool was piloted in the Cape Winelands and Overberg districts at district hospitals or community health centres wherever there was a permanent family physician. An email explaining the research and the piloting process, as well as asking whether or not they would like to participate, was sent to all the family physicians, with the exception of the three family physicians based at Worcester Regional Hospital. They were excluded because they were not working in the district health services. All of the family physicians who were included agreed to participate in the study. The family physicians were then asked to identify ten to fifteen people in any of the above mentioned categories, namely: managers, doctors, nurses and other health professionals, who work alongside them and who would be willing to complete the tool. No specification was made as to the number of respondents selected per category. However, it was stipulated that at least one manager should be included in the evaluation.

Three open-ended questions were included at the end of the validated tool to again get feedback from the respondents regarding the content and quality of the tool. Each family physician received a large envelope containing fifteen questionnaires together with a letter explaining their purpose, each placed in a smaller envelope. The family physicians were given the responsibility of handing out the questionnaires to the people that they had selected. The respondents were instructed to complete the questionnaire, place and seal it in the small envelope provided and hand it back to the family physician, who then collected them and placed them back in the large envelope. The questionnaire did not ask the respondents to give their name. The sealed questionnaires were then personally collected from the family physicians by the researcher. This was done in an attempt to ensure confidentiality of the respondents.

### Testing reliability

The results of the pilot study were analysed with the help of a statistician from the Centre for Statistical Consultation at Stellenbosch University. Answers to the individual statements were substituted for numbers (i.e. 1 = strongly disagree; 2 = disagree; 3 = agree; and 4 = strongly agree) according to the Likert scale. The options for ‘not part of their work’ and ‘unable to answer’ were not included in this analysis, as these meant that the respondents were unable to make a useful assessment. A Cronbach Alpha was then calculated to determine the reliability of the tool and the individual statements. This is a number between 0 and 1, with values close to 1 indicative of better reliability/internal consistency, as indicated below.

- 0.00 – 0.39 = poor reliability
- 0.40 – 0.59 = average reliability
- 0.60 – 0.79 = good reliability
- > = 0.80 = excellent reliability

A Cronbach Alpha was calculated for each family physician role by analysing all the statements for all the family physicians. Furthermore, the reliability of each statement was analysed by showing what the Cronbach Alpha would have been had the statement been deleted or omitted from the analysis and if a statement was not performing well then the Cronbach Alpha would increase.

### Analysing results on the impact of the family physicians

The results were further analysed and the mean score for each family physician and for each role was calculated based on the Likert scale from 1 to 4. The mean score could then be interpreted as:

- Score < 2: No impact in this area
- Score ≥ = 2 but < 3: Little impact in this area
- Score ≥ = 3 but < 3.5: Moderate impact in this area
- Score ≥ = 3.5: High impact in this area

A comparison was made between the overall perceived impact of the family physicians in the various roles. The post-hoc Bonferroni test was used to see where any statistically significant difference occurred between the various roles. A comparison was also made between the perceived impact of family physicians in the Cape Winelands and Overberg districts.

Lastly, the small amount of qualitative feedback from the respondents was also analysed. A single researcher collated all the comments and organised them into categories. Comments that were relevant to the study objectives are reported on in the results section.

### Ethical considerations

Ethical approval was obtained from the Health Research Ethics Committee at Stellenbosch University (N11/10/012) and permission to conduct the research from the Department of Health. Appropriate written consent was obtained from the participants.

## Results

### Content and construct validation of the tool

Feedback on the draft tool was received from 10 experts: 2 training programme managers; 2 district managers; 2 training complex co-ordinators; 3 researchers and the director of the Health Impact Assessment Directorate. The following key changes were made:

- The name of the tool was changed to Family Physician Impact Evaluation Tool as this was more congruent with its purpose.
- Some of the information collected on the respondents was thought to be unnecessary and was removed, their job title and therefore relationship to the family physician was retained.
- Two response options for each statement were added. These were ‘unable to answer’ and ‘not part of their work’. ‘Unable to answer’, implied you did not see this aspect of the work and could not express an opinion. ‘Not part of their work’, implied that the question was not applicable to their current job description.
- Grammatical changes were made, as well as the rephrasing of certain statements. Examples were added to clarify statements and help respondents interpret them correctly.
- A few additional statements were added such as “The Family Physician is available for consultation and is not taken up by too many non-clinical duties”.
- Significant changes were made to the Supervisor and Trainer section, through the combination and clarification of statements.

### Piloting of the tool

The final tool was then piloted and tested for reliability. Eight family physicians participated in the piloting of the tool, four of whom were from the Winelands and four from the Overberg district. In total, there were 95 respondents of which one respondent evaluated the wrong person and therefore was excluded. Fourteen of the respondents failed to indicate their job title, but were nonetheless included in the analysis. Table 1 gives a breakdown of the respondents and their job descriptions:

**Table 1:** **Profile of respondents (N = 94)**

**Job title**	**n (%)**
Hospital/district/sub-district managers	10 (10.5)
Nursing managers	5 (5.3)
Family physicians	9 (9.5)
Family medicine registrars	1 (1.0)
Medical officers	18 (19.1)
Community service doctors	6 (6.3)
Interns	1 (1.0)
Nursing sisters/staff nurses	10 (10.6)
Radiographers	4 (4.2)
Physiotherapists	3 (3.1)
Pharmacists	2 (2.1)
Social workers	1 (1.0)
Dieticians	1 (1.0)
Other	9 (9.5)
Unspecified	14 (14.8)

### Reliability of the tool

The Cronbach Alpha for each main role was above 0.8 (see Table 2), which means that the internal consistency/ reliability of questions combined for each main role was excellent. There was also very little change in the Cronbach Alpha after the omission of individual questions, with the greatest increase being only 0.04. None of the individual questions were therefore considered unreliable.

**Table 2:** **Cronbach alpha for each family physician role**

**Family Physician roles**	**Cronbach Alpha**
Care provider	0.84
Consultant	0.82
Leader and champion of clinical governance	0.94
Clinical trainer and supervisor	0.92
Capacity builder	0.86
Leader and champion of COPC	0.90

### Evaluation of the family physicians’ impact

**Figure 2:**
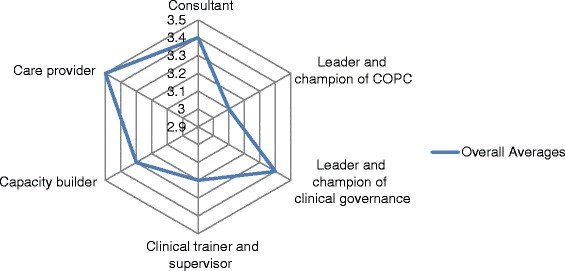
**The overall perceived impact of Family Physicians in the Cape Winelands and Overberg districts.**

The mean scores for each of the roles were all above 3.0 as shown in Figure 2. The results were as follows: Care provider = 3.5, Consultant = 3.4, Leader and champion of clinical governance = 3.4, Capacity builder = 3.3, Clinical trainer and supervisor = 3.2 and Leader and champion of COPC = 3.1. The scores suggest a moderate perceived impact in all areas and a high perceived impact in the area of clinical care provision.

The family physicians’ perceived impact in their role as leader and champion of COPC was significantly lower than their impact as a care provider (p < 0.001), consultant (p = 0.001) and leader and champion of clinical governance (p = 0.005). Their perceived impact as clinical trainer and supervisor was also significantly lower than their perceived impact as care provider (p = 0.018). There was no statistically significant difference between perceived impact as a capacity builder and any of the other roles.

Tables 3, 4, 5, 6, 7, and 8 present the mean scores for the individual competencies that relate to the overall assessment of each role.

**Table 3:** **Mean scores for competencies related to the impact of family physicians as care providers**

**Competency for the role**	**Mean score**
The family physician is competently able to manage patients with HIV at a primary care level.	3.6
The family physician is able to competently diagnose TB and to initiate treatment.	3.7
The family physician is able to competently manage patients with non-communicable diseases, e.g. hypertension/diabetes/asthma.	3.6
The family physician is able to competently manage women in labour and deal with obstetric and gynaecological emergencies.	3.5
The family physician is able to competently manage children with common childhood conditions e.g. malnutrition/diarrhoeal disease/lower respiratory tract infections.	3.6
The family physician is able to competently stabilise patients with poly-trauma.	3.4
The family physician is able to competently manage patients with common medical emergencies and conditions.	3.6
The family physician is able to competently manage patients with common surgical and orthopaedic emergencies and conditions.	3.4
The family physician is able to recognise and manage patients with mental illness and refer appropriately, and where appropriate, to begin treatment.	3.5
The family physician is able to competently give anaesthetic/sedation to patients who are a low anaesthetic risk.	3.3
The family physician is able to competently manage sexual assault or intimate partner violence.	3.5

**Table 4:** **Mean scores for competencies related to the impact of family physicians as consultants**

**Competency for the role**	**Mean score**
I feel more supported in my clinical work knowing that there is a family physician on site.	3.5
The family physician is a role model for patient-centred clinical care.	3.7
When dealing with a patient, the family physician often asks about their family and context.	3.6
The presence of the family physician has decreased unnecessary referrals to level 2 and 3 hospitals.	3.3
The family physician often sees patients with more complicated conditions referred by Clinical Nurse Practitioners/Doctors in primary care.	3.3
The family physician often sees patients with more complicated conditions in the hospital wards.	3.1
The family physician knows and understands the limitations as a consultant (i.e. knows when to refer or ask for help appropriately).	3.6
The family physician performs outreach to other clinics or health centres.	3.4
The family physician remains up to date with the latest guidelines and evidence.	3.5
The family physician is available for consultation and is not taken up by too many non-clinical duties.	3.0

**Table 5:** **Mean scores for competencies related to the impact of family physicians as leaders and champions of clinical governance**

**Competency for the role**	**Mean score**
The family physician creates a positive climate at work that motivates/supports staff to do their best.	3.5
The family physician promotes increased levels of teamwork through his/her leadership style.	3.4
The family physician displays skill in resolving conflict productively.	3.3
The family physician handles his/her own stress and pressure well and is sensitive to the needs of staff with regards to handling their stress.	3.3
The family physician has a calming influence on others.	3.2
The family physician is concerned with the personal wellbeing of his/her staff.	3.5
The family physician is continuously trying to improve systems to provide better quality of care i.e. through quality improvement cycles, morbidity and mortality meetings, clinical management meetings, functional business meetings etc.	3.5
The family physician promotes or engages in health prevention strategies i.e. cervical or breast cancer screening programmes etc.	3.2
The family physician places high emphasis on the involvement of the multidisciplinary team (i.e. nurses/ occupational therapists/ physiotherapists/ social worker etc.) in clinical decision- making.	3.5
The family physician creates or helps to drive plans to further develop your hospital/clinic.	3.4
The family physician improves the patients’ experience of care at this facility i.e. tries to reduce waiting times etc.	3.4

**Table 6:** **Mean scores for competencies related to the impact of family physicians as clinical trainers and supervisors**

**Competency for the role**	**Mean score**
The family physician contributes to the training of interns or community service doctors.	3.4
The family physician contributes to the training of family medicine registrars, e.g. through educational meetings, observed consultations of registrars or by supervising their course work.	3.1
The family physician contributes to the training of undergraduate students, e.g. through giving tutorials, bedside teaching, or supervising their projects.	3.3
The family physician is involved in the assessment of under- and post-graduate students e.g. portfolio, oral and clinical assessments.	3.2
Having students supervised by the family physician has a positive impact on the quality of care at the facility e.g. through student projects.	3.2
Having students supervised by the family physician has a positive impact on the learning environment at the facility e.g. more academic meetings and greater academic influence.	3.2

**Table 7:** **Mean scores for competencies related to the impact of family physicians as capacity builders**

**Competency for the role**	**Mean score**
The family physician promotes the continuous professional development of his/her staff by organizing or facilitating CPD activities or by creating space for staff to attend courses/workshops.	3.2
The family physician builds capacity through delegating tasks and responsibilities while giving support.	3.3
The family physician is interested in the development of the staff as professionals and as people.	3.4
The family physician is easily approachable.	3.5
The family physician provides constructive feedback to staff on professional development and openly discusses mistakes in a constructive manner.	3.4
My clinical practice has improved because of the presence of a family physician.	3.2
The family physician helps to make the CHC/DH a place where learning happens on a daily basis, e.g. calls people to see an interesting patient, puts up articles for others to read, encourages one to discuss mistakes.	3.2

**Table 8:** **Mean scores for competencies related to the impact of family physicians as leaders and champions of COPC**

**Competency for the role**	**Mean score**
The family physician is aware of the health problems of the local community/district.	3.5
The family physician has a vision for health promotion in the community served and has communicated this to the staff.	3.4
The family physician is currently engaged in/supporting health promotion in the community served.	3.3
The family physician engages with other community-based resources and services i.e. NGOs, churches, local government.	3.0
The family physician engages with local community leaders.	2.7
The family physician is involved in strengthening community-based services i.e. joining, training, collaborating or supporting community health care workers and home-based carers.	3.1
The family physician has a vision beyond the hospital/clinic to making a positive impact on the health of the community served and has communicated this to the staff.	3.2
The family physician manages patients in a step-down or rehabilitation facility.	2.6
The family physician is involved in strengthening/improving a step-down or rehabilitation facility.	2.7

The highest scores (≥ 3.5) indicated that the family physicians were seen as highly competent clinicians across the whole burden of disease. They were seen as role models for an evidence-based, holistic and patient-centred approach and other health workers felt more confident knowing that a family physician was available to consult. They were seen to foster a collaborative and multi-disciplinary approach to patient care. They were perceived to know their limitations and when referral was necessary. Health workers also felt that family physicians created a positive organisational culture in which staff felt more engaged and supported. They were perceived to be champions of clinical governance and continuously trying to improve the quality of care.

Family physicians were perceived to have a moderate and positive impact (3.0-3.4) in terms of poly-trauma, surgical and anaesthetic skills. They were seen to reduce referrals to secondary and tertiary levels by seeing more complicated patients in primary care and district hospitals. They were perceived to provide outreach to primary care services and to be available for consultation. They were seen as developing preventative and promotive activities.

They were also seen as making a moderate contribution to the training of interns, medical students and registrars; and to the creation of a culture of learning and innovation. They were reported to have a vision for community-orientated primary care and to be making some progress in the realisation of this through strengthening community-based services, encouraging health promotion and engaging with local non-government organisations and resources.

The lowest scores (< 3.0) implied that family physicians were perceived to have little impact in terms of relating to local community leaders and in terms of providing care at step-down or rehabilitative facilities.

Further results showed no statistically significant difference in the perceived impact of family physicians between the Cape Winelands and Overberg districts.

### Results of the qualitative feedback from the piloting

Two of the family physicians felt that their current job positions meant that they were not expected to fulfil all of the expected roles. The one felt that some of the questions were not relevant to their current job description and the other said they had a 5/8 post and did not work in a district hospital which meant that their scope of practice was limited. Another respondent commented that the family physician may not be involved in all of the roles being evaluated.

Some respondents felt that they would have liked to give more qualitative feedback to illustrate their answers. One of the respondents at the same district hospital felt that there also needed to be a question about family physicians performing tasks or duties beyond their expected roles.

## Discussion

This study has resulted in a valid and reliable tool that can be used to evaluate the impact of family physicians in the South African public sector district health services.

The evaluation of the family physician’s impact was not the primary purpose of this study and will need to be studied in a larger sample. Nevertheless the findings were interesting in showing that in the Winelands and Overberg districts the highest impact was in the area of clinical care provision, which is one of the primary objectives of the now formalised family medicine registrar programme [21]. These family physicians were also perceived to be making a moderate impact in the areas of clinical leadership and governance, and as consultants to the rest of the healthcare team, which according to the World Health Organization of family doctors, is where family physicians are expected to make an important impact [10]. Their lowest impact was in the area of championing COPC, which is an important focus of the government’s policy on re-engineering primary care and in some areas where COPC has been more systematically implemented the supportive role of the family physician has been more clearly defined and operationalised [19,26]. Other studies support the view that in Africa the commitment to COPC is still largely aspirational [14,15]. Family physicians also had a lower impact in their role as clinical trainers and supervisors and the tension between service delivery and clinical training of students has been noted elsewhere [27].

A possible limitation was that the family physicians were given the responsibility of selecting people to evaluate them. However, one could argue that the family physicians have a much better idea of the people working alongside them and who could give an accurate assessment, and should therefore select the respondents themselves. The available pool of relevant people at the facilities is also relatively small and it would be difficult to only select people with a more positive viewpoint. In future it may be better to specify a number of respondents from each category of healthcare worker. A further adaptation could be to select respondents randomly from the total pool of eligible people in each category provided by the family physician.

Although confidentiality was maintained, the family physicians were given the responsibility of handing out and collecting the tool from the respondents. This could have affected the way the respondents answered the tool, which is another potential limitation. In some studies, if funding and distances permit, it may be possible to disseminate and collect the questionnaires by means of a research assistant.

Although developed in the Western Cape the tool should be applicable to South Africa as the roles on which it is based were agreed to nationally and the context is broadly similar. The health system in some other provinces is however less developed and there may be differences in the emphasis put on different roles.

Other key amendments to the use of the tool derived from the qualitative feedback were clearer explanations of the options “Not part of family physician’s work” and “I do not see this aspect of the family physician’s work” so that respondents could express where they felt their job descriptions differed from the roles outlined in the tool. In addition space has been added for qualitative feedback on the answers. The final tool has been uploaded as an Additional file 1.

Now that the impact evaluation tool is developed, validated and reliable, it can be used to evaluate family physicians on a larger scale. Further research is planned in the Western Cape as well as nationally to utilise the tool for this purpose.

Some managers query whether a family physician with postgraduate training adds significant value to the health services when compared with career medical officers without such formal training. The evaluation tool does not include any comparison of the impact of the family physician relative to medical officers and future research could add additional questions on this issue or use the tool to compare the two groups.

## Conclusion

The current drive to strengthen our district health system has drawn a lot of attention to the role and impact of the family physician. A family physician impact evaluation tool, has been developed, validated and proved reliable. The tool can now be used to assess impact on a larger scale and hopefully provide valuable and usable information to managers and policy makers. The piloting of this tool in the Western Cape revealed that family physicians were perceived to be making a high/moderate impact in the areas of clinical care provision, leadership and championing of clinical governance. However, they had less of an impact in the areas of clinical training and supervising, capacity building and championing of community-orientated primary care.

## Availability of supporting data

The raw data used to calculate the results is stored at the Division of Family Medicine and Primary Care and can be made available on request once all identifiers have been removed.

## Additional file

Additional file 1:
**The raw data used to calculate the results is stored at the Division of Family Medicine and Primary Care and can be made available on request once all identifiers have been removed.**
