# Supplementary material for: Development of a family physician impact assessment tool in the district health system of the Western Cape Province, South Africa
Source: BMC Fam Pract. 2014 Dec 12;15:204. doi: 10.1186/s12875-014-0204-7 (PMC4276011; doi:10.1186/s12875-014-0204-7)
Supplement: Additional file 1: — The raw data used to calculate the results is stored at the Division of Family Medicine and Primary Care and can be made available on request once all identifiers have been removed. [file 12875_2014_204_MOESM1_ESM.docx]

**FAMILY PHYSICIAN**

**IMPACT EVALUATION TOOL**

| **PARTICIPANT INFORMATION** |
| --- |
| **Name of family physician being evaluated:** |
| **What is your position (tick the appropriate box):**   \| District Manager \| Sub-District Manager \| Hospital Manager/Superintendent \| Family Physician \| \| --- \| --- \| --- \| --- \| \| Family Medicine Registrar \| Medical Officer \| Community Service Doctor \| Intern \| \| Nursing Manager \| Nursing Sister/Staff Nurse \| Audiologist \| Dietician \| \| Pharmacist \| Physiotherapist \| Psychologist \| Radiographer \| \| Occupational Therapist \| Social Worker \| Speech Therapist \| Community Health Worker \| \| Other (please specify): \| \| \| \| |

| **TOOL GUIDELINES** |
| --- |
| Kindly complete this tool, by responding to the statements below and ticking the appropriate box.  **The following scale is used:**     \| Strongly  Disagree \| Disagree \| Agree \| Strongly  Agree \| Not part of the family physician’s work/job description \| I don’t see this aspect of the family physician’s work \| \| --- \| --- \| --- \| --- \| --- \| --- \|   **Note:** Please use the space below each set of statements to clarify your answers should you wish to do so, and for any additional comments. |

| **CONSULTANT** | | | | | | |
| --- | --- | --- | --- | --- | --- | --- |
|  | Strongly  Disagree | Disagree | Agree | Strongly  Agree | Not part of family physician’s work/job description | I don’t see this aspect of the family physician’s work |
| 1. I feel more supported in my clinical work knowing that there is a family physician on site. |  |  |  |  |  |  |
| 1. The family physician is a role model for patient-centred clinical care. |  |  |  |  |  |  |
| 1. When dealing with a patient, the family physician often asks about their family and context. |  |  |  |  |  |  |
| 1. The presence of the family physician has decreased unnecessary referrals to level 2 and 3 hospitals. |  |  |  |  |  |  |
| 1. The family physician often sees patients with more complicated conditions referred by Clinical Nurse Practitioners/Doctors in primary care. |  |  |  |  |  |  |
| 1. The family physician often sees patients with more complicated conditions in the hospital wards. |  |  |  |  |  |  |
| 1. The family physician knows and understands the limitations as a consultant (i.e. knows when to refer or ask for help appropriately). |  |  |  |  |  |  |
| 1. The family physician performs outreach to other clinics or health centres. |  |  |  |  |  |  |
| 1. The family physician remains up to date with the latest guidelines and evidence. |  |  |  |  |  |  |
| 1. The family physician is available for consultation and is not taken up by too many non-clinical duties. |  |  |  |  |  |  |
| Comments | | | | | | |

| **LEADER AND CHAMPION OF COMMUNITY ORIENTATED PRIMARY CARE** | | | | | | |
| --- | --- | --- | --- | --- | --- | --- |
|  | Strongly  Disagree | Disagree | Agree | Strongly  Agree | Not part of family physician’s work/job description | I don’t see this aspect of the family physician’s work |
| 1. The family physician is aware of the health problems of the local community/district. |  |  |  |  |  |  |
| 2. The family physician has a vision for health promotion in the community served and has communicated this to the staff. |  |  |  |  |  |  |
| 3. The family physician is currently engaged in/supporting health promotion in the community served. |  |  |  |  |  |  |
| 4. The family physician engages with other community-based resources and services i.e. NGOs, churches, local government. |  |  |  |  |  |  |
| 5. The family physician engages with local community leaders. |  |  |  |  |  |  |
| 6. The family physician is involved in strengthening community-based services i.e. joining, training, collaborating or supporting community health care workers and home-based carers. |  |  |  |  |  |  |
| 7. The family physician has a vision beyond the hospital/clinic to making a positive impact on the health of the community served and has communicated this to the staff. |  |  |  |  |  |  |
| 8. The family physician manages patients in a step-down or rehabilitation facility. |  |  |  |  |  |  |
| 9. The family physician is involved in strengthening/improving a step-down or rehabilitation facility. |  |  |  |  |  |  |
| Comments | | | | | | |

| **LEADER AND CHAMPION OF CLINICAL GOVERNANCE** | | | | | | | | | | | | | |
| --- | --- | --- | --- | --- | --- | --- | --- | --- | --- | --- | --- | --- | --- |
|  | | Strongly  Disagree | | Disagree | | Agree | | Strongly  Agree | | Not part of family physician’s work/job description | | I don’t see this aspect of the family physician’s work | |
| 1. The family physician creates a positive  climate at work that motivates/supports staff to do their best. | |  | |  | |  | |  | |  | |  | |
| 2. The family physician promotes increased levels of teamwork through his/her leadership style. | |  | |  | |  | |  | |  | |  | |
| 3. The family physician displays skill in resolving conflict productively. | |  | |  | |  | |  | |  | |  | |
| 4. The family physician handles his/her own stress and pressure well and is sensitive to the needs of staff with regards to handling their stress. | |  | |  | |  | |  | |  | |  | |
| 5. The family physician has a calming influence on others. | |  | |  | |  | |  | |  | |  | |
| 6. The family physician is concerned with the personal wellbeing of his/her staff. | |  | |  | |  | |  | |  | |  | |
| 7. The family physician is continuously trying to improve systems to provide better quality of care i.e. through quality improvement cycles, morbidity and mortality meetings, clinical management meetings, functional business meetings etc. | |  | |  | |  | |  | |  | |  | |
| 8. The family physician promotes or engages in health prevention strategies i.e. cervical or breast cancer screening programmes etc. | |  | |  | |  | |  | |  | |  | |
| 9. The family physician places high emphasis on the involvement of the multidisciplinary team (i.e. nurses/ occupational therapists/ physiotherapists/ social worker etc.) in clinical decision-making. | |  | |  | |  | |  | |  | |  | |
| 10. The family physician creates or helps  to drive plans to further develop your   hospital/clinic. | |  | |  | |  | |  | |  | |  | |
| 11. The family physician improves the patients’ experience of care at this facility i.e. tries to reduce waiting times etc. | |  | |  | |  | |  | |  | |  | |
| Comments | | | | | | | | | | | | | |
| **CLINICAL TRAINER AND SUPERVISOR** | | | | | | | | | | | | |  |
|  | Strongly  Disagree | | Disagree | | Agree | | Strongly  Agree | | Not part of family physician’s work/job description | | I don’t see this aspect of the family physician’s work | |  |
| 1. The family physician contributes to the training of interns or community service doctors. |  | |  | |  | |  | |  | |  | |  |
| 2. The family physician contributes to the training of family medicine registrars, e.g. through educational meetings, observed consultations of registrars or by supervising their course work. |  | |  | |  | |  | |  | |  | |  |
| 3. The family physician contributes to the training of undergraduate students, e.g. through giving tutorials, bedside teaching, or supervising their projects. |  | |  | |  | |  | |  | |  | |  |
| 4. The family physician is involved in the assessment of under- and post-graduate students e.g. portfolio, oral and OSCE assessments. |  | |  | |  | |  | |  | |  | |  |
| 5. Having students supervised by the family physician has a positive impact on the quality of care at the facility e.g. through student projects. |  | |  | |  | |  | |  | |  | |  |
| 6. Having students supervised by the   family physician has a positive   impact on the learning environment   at the facility e.g. more academic   meetings and greater academic   influence. |  | |  | |  | |  | |  | |  | |  |
| Comments | | | | | | | | | | | | |  |

| **CAPACITY BUILDER** | | | | | | |
| --- | --- | --- | --- | --- | --- | --- |
|  | Strongly  Disagree | Disagree | Agree | Strongly  Agree | Not part of family physician’s work/job description | I don’t see this aspect of the family physician’s work |
| 1. The family physician promotes the continuous professional development of his/her staff by organizing or facilitating CPD activities or by creating space for staff to attend courses/workshops. |  |  |  |  |  |  |
| 2. The family physician builds capacity through delegating tasks and responsibilities while giving support. |  |  |  |  |  |  |
| 3. The family physician is interested in the development of the staff as professionals and as people. |  |  |  |  |  |  |
| 4. The family physician is easily approachable. |  |  |  |  |  |  |
| 5. The family physician provides constructive feedback to staff on professional development and openly discusses mistakes in a constructive manner. |  |  |  |  |  |  |
| 6. My clinical practice has improved because of the presence of a family physician. |  |  |  |  |  |  |
| 7. The family physician helps to make the CHC/DH a place where learning happens on a daily basis, e.g. calls people to see an interesting patient, puts up articles for others to read, encourages one to discuss mistakes. |  |  |  |  |  |  |
| Comments | | | | | | |

| **CARE PROVIDER** | | | | | | |
| --- | --- | --- | --- | --- | --- | --- |
|  | Strongly  Disagree | Disagree | Agree | Strongly  Agree | Not part of family physician’s work/job description | I don’t see his aspect of the family physician’s work |
| 1. The family physician is competently able to manage patients with HIV at a primary care level. |  |  |  |  |  |  |
| 2. The family physician is able to competently diagnose TB and to initiate treatment. |  |  |  |  |  |  |
| 3. The family physician is able to competently manage patients with non-communicable diseases, e.g. hypertension/diabetes/asthma. |  |  |  |  |  |  |
| 4. The family physician is able to competently manage women in labour and deal with obstetric and gynaecological emergencies. |  |  |  |  |  |  |
| 5. The family physician is able to competently manage children with common childhood conditions e.g. malnutrition/diarrhoeal disease/lower respiratory tract infections. |  |  |  |  |  |  |
| 6. The family physician is able to competently stabilise patients with poly-trauma. |  |  |  |  |  |  |
| 7. The family physician is able to competently manage patients with common medical emergencies and conditions. |  |  |  |  |  |  |
| 8. The family physician is able to competently manage patients with common surgical and orthopaedic emergencies and conditions. |  |  |  |  |  |  |
| 9. The family physician is able to recognise and manage patients with mental illness and refer appropriately, and where appropriate, to begin treatment. |  |  |  |  |  |  |
| 10. The family physician is able to competently give anaesthetic/sedation to patients who are a low anaesthetic risk. |  |  |  |  |  |  |
| 1. The family physician is able to competently manage sexual assault or intimate partner violence. |  |  |  |  |  |  |
| Comments | | | | | | |
